# 

**Published:** 1878-04

**Authors:** 


### CONTENTS.

PAGE.

**CONTRIBUTIONS.**

Abuses of the Stomach. BEN. H. PRATT ...... ...... 1

Diathesis P. HAROLD HAYES, M. D. ............... 5

Reuben. AUSBURN TOWNER ......................... 7

**MEDICAL ITEMS AND NEWS.**

Hydrophobia—Iodized Cod Liver Oil—Nausea and Vomiting—Cholera—Asthma—Testing Purity of Chloroform—Eucalyptus as a Local Anæsthetic—Chloral dangerous in Delirium Tremens—Boils—Total Abstinence—Growth of Hair after Death—Vance's Cream for Chilblains—Death from chewing Tobacco—Foreign Bodies in the Throat—Cough—Migraine—Injection of Liquids into Bladder—Gonorrhœa—Digestion of Cod Liver Oil—Prickly Heat—Obstinate Diarrhœa—Living Insects in the Ear—New Counter-Irritant—Typhoid Fever—To Blister Quickly—Ciliary Neuralgia—Bright's Disease in apparently healthy subjects—Danger of Ether—Needle found in the Brain—To lessen quantity of Quinine—Epilepsy—Orchitis—Death from Carious Tooth—Foreign Bodies in the Stomach—Pathological Sweating—Glycosuria—Intestinal Gas and Flatulent Dyspepsia—Tearless Madness—Perpetual Convulsions—Hypodermic use of Ether in Labor—Home made Mineral Waters—Scrofulous Diseases—Lister's Antiseptic Medicaments—Diphtheria—Ozæna—Pimply-Face Acne—Ergotine for Hypodermic injections in Metrorrhagia ............... 10 to 21

**OUR CORRESPONDENCE.**

Eleven Letters with Answers ................... 21 to 23

**HOUSEHOLD HINTS AND RECIPES.**

Ink, red—Cement for Porcelain—Iron, to silver—Oil of Eggs—Cement for Rubber—Ink, Black—Mince Pies—Kerosene Liniment—Canine Distemper—Liquid Glue—Artificial Milk—Vermin on Poultry—Wash for Hands—Flowers, to crystallize ........................ 23 to 25

**EDITORIAL.**

Injuries to the Eyeball ........................ 25
Medical Advertisements ......................... 25
On Horseback ................................... 26

**EDITORIAL CHIT-CHAT.**

Volume XIV—Corrected—Fine Horse—Trichinæ—Protective Tariff—Horse Frighteners—No more Dead-Heads—Title Page—June Vacation—The Difference—Ingersoll—Park Church Choir—Parvules — Acorns — Children's Exposure — Plant Flowers—Spring Time—The Great English Physician—Reuben—Phonograph ................ 28 to 31

